# Molecular characterization of subcutaneous panniculitis-like T-cell lymphoma reveals upregulation of immunosuppression- and autoimmunity-associated genes

**DOI:** 10.1186/s13023-014-0160-2

**Published:** 2014-11-12

**Authors:** Pilvi Maliniemi, Sonja Hahtola, Kristian Ovaska, Leila Jeskanen, Liisa Väkevä, Kirsi Jäntti, Rudolf Stadler, David Michonneau, Sylvie Fraitag, Sampsa Hautaniemi, Annamari Ranki

**Affiliations:** Department of Dermatology and Allergology, University of Helsinki and Helsinki University Central Hospital, Helsinki, Finland; Systems Biology Laboratory, Institute of Biomedicine and Genome-Scale Biology Program, University of Helsinki, Helsinki, Finland; Johannes-Wesling-Klinikum Minden, Akademisches Lehrkrankenhaus der Medizinischen Hochschule Hannover, Minden, Germany; Institut Pasteur, Département d’immunologie, Equipe Dynamique des réponses immunes, 25 rue du Docteur Roux, 75015 Paris, France; Service d’anatomie et de cytologie pathologiques, Hôpital Necker-Enfants-Malades, AP-HP, 149, rue de Sèvres, 75743 Paris Cedex 15, France

**Keywords:** Subcutaneous panniculitis-like T-cell lymphoma, IDO-1, Immunosuppressive tumor microenvironment, Th1-type

## Abstract

**Background:**

Subcutaneous panniculitis-like T cell lymphomas represent a rare and difficult to diagnose entity of cutaneous T cell lymphomas. SPTL affects predominantly young adults and presents with multifocal subcutaneous nodules and frequently associated autoimmune features. The pathogenesis of SPTL is not completely understood.

**Methods:**

The aim of this study was to unravel molecular pathways critical to the SPTL pathogenesis. Therefore, we analyzed 23 skin samples from 20 newly diagnosed SPTL patients and relevant control samples of adipose and non-malignant panniculitis tissue by using gene expression microarray, quantitative PCR, and two-colour immunohistochemistry.

**Results:**

Interestingly, indoleamine 2,3-dioxygenase (*IDO-1*), an immunotolerance-inducing enzyme, was among the most highly overexpressed genes in all comparisons. The expression of Th1-specific cytokines, known to be associated with autoimmune inflammation (*i.e. IFNG, CXCR3, CXCL9, CXCL10, CXCL11,* and *CCL5*), were also significantly increased. Confirmed using immunohistochemistry, the morphologically malignant lymphocytes expressed CXCR3 and CXCL9. IDO-1 expression was found both in some morphologically malignant lymphocytes rimming the adipocytes and in surrounding CD11c^−^ CD68^−^ cells but not in CD11c^+^ dendritic cells in the microenvironment. The proportion of FoxP3+ cells in SPTL exceeded that in the benign panniculitis samples.

**Conclusions:**

Our results indicate that the up regulation of the tolerogenic IDO-1 together with the up regulation of IFNG, CXCR3 ligands, and CCL5 are features of SPTL lesions. We anticipate that the IFNG-inducible IDO-1 expression contributes to the formation of an immunosuppressive microenvironment, favorable for the malignant T cells. This study provides a relevant molecular basis for further studies exploring novel therapeutic means for subcutaneous T cell lymphoma.

**Electronic supplementary material:**

The online version of this article (doi:10.1186/s13023-014-0160-2) contains supplementary material, which is available to authorized users.

## Background

Subcutaneous panniculitis–like T-cell lymphoma (SPTL, ORPHA86884) represents a rare entity of T-cell lymphomas. The pathomechanism of SPTL is not known but mostly SPTL has a favourable prognosis and it responds to non-aggressive (immunosuppressive) therapy. The most recent WHO-EORTC classification, together with the EORTC Cutaneous Lymphoma Group Report, confine SPTL to subcutaneous lymphomas with an α/β T-cell phenotype and neoplastic T-cells expressing CD3, CD8 and cytotoxic proteins (GZMB, TIA-1, perforin) [1].

In a recent joint study by the EORTC Cutaneous Lymphoma Group [1], the main clinical, histopathological, and prognostic features of SPTL were defined in a long-term collection of 83 European SPTL cases. The clinical characteristics of SPTL include multifocal, nodular skin lesions or deeply seated plaques involving the legs, arms, and trunk, and less commonly the face. Ulcerations are uncommon. B-symptoms (fever, weight loss) or laboratory abnormalities are encountered in about half of the cases. The most common laboratory abnormalities include various cytopenias and elevated liver function tests. Autoimmune diseases are common among SPTL patients [2,3], as 20% of the patients in the European cohort had an associated autoimmune disorder, most commonly systemic lupus erythematosus (SLE) and some cases were first misdiagnosed as lupus panniculitis (lupus profundus) [1]. Histologically, SPTL is characterized by lobular panniculitis with a subcutaneous atypical lymphocyte proliferation rimming the adipocytes. The characteristic immunophenotype of the neoplastic cells is CD3+, CD4-, CD8+, CD30-, and CD56-. Cytotoxic proteins (TIA-1, GZMB, perforin) are usually strongly expressed. The prognosis of SPTL is favourable, with a 5-year survival of 91% (82% if hemophagocytic syndrome is present).

No previous studies on the pathomechanism of SPTL exist, possibly due to the fact that the incidence of SPTL is 0.9% of all CTCLs [4] and therefore it is extremely difficult to gain access to fresh subcutaneous fat tissue biopsies prior to treatment in these rare but young patients. We now report on the gene expression profile of fresh, untreated, and dissected SPTL skin lesions with Human Exon 1.0 microarray, and subsequent confirmatory quantitative RT-PCR, and immunohistological results on the cellular origin of the deregulated genes. For the first time, the molecular signature characteristic of inflammation in SPTL is revealed.

## Methods

### Patient material

**Figure 1:**
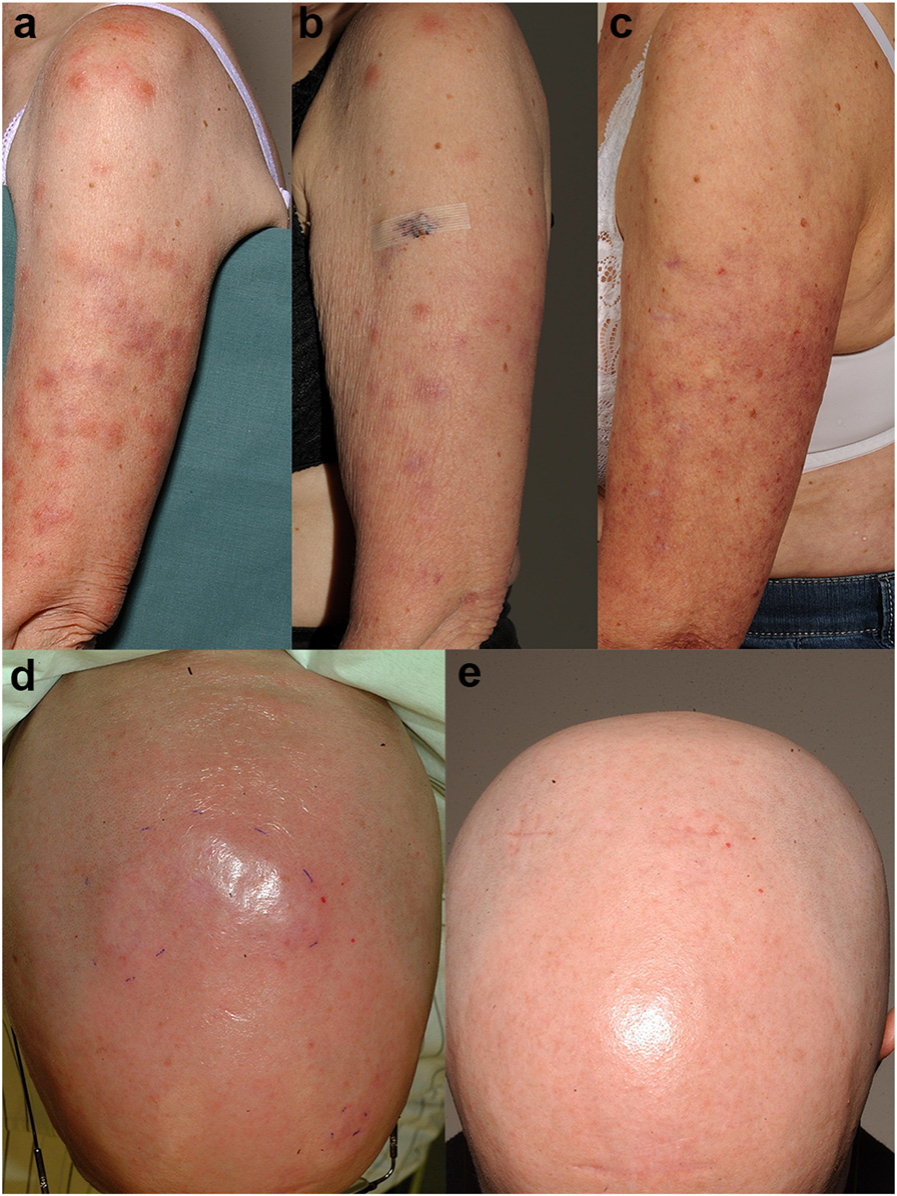
**Clinical presentation of SPTL lesions.** Representative SPTL lesions before **(a, d)**, during **(b)**, and after **(c, e)** systemic steroid +/− methotrexate treatment. For treatment details see Table 1 (case 3 and 2, respectively).

Altogether, the study included 20 SPTL patients from three different European nations (Table 1), of whom four pre-treatment skin samples were analyzed by gene expression microarray, five samples by quantitative RT-PCR, and 23 samples by immunohistochemistry (IHC). Of one of the patients included in the array analysis, also an affected inguinal lymph node was biopsied and available for immunohistochemistry. Additionally, three Finnish SPTL patients were followed during treatment with oral prednisolone and low-dose methotrexate (Table 1) and altogether, a set of eight follow-up skin samples were obtained and analysed with the microarray. The demographic details of the patients are given in Table 1 and clinical presentation of lesions in Figure 1.

**Table 1:** **Clinical presentation, treatment and outcome of the 20 subcutaneous panniculitis-like T-cell lymphoma (SPTL) patients studied**

**Case**	Sex and age*	**First clinical presentation**	Clinical picture, concomitant diseases*	**Abnormal laboratory findings**	**Therapy**	**Outcome**	**PE**
1	F15	In 2006 first sub-cutaneous lesion laterally on trunk, a year later ca. 50 widespread subcutaneous nodules, SPTL diagnosis in 05/2008	No B-symptoms	CT normal, mild splenomegaly (diam. 13 cm)	06/2008 prednisolone	CR since 06/2010; 07/2011 a solitary MF lesion	
			Otherwise healthy	No ANA/ ENA/DNA antibodies	60 mg/d (2 weeks), slowly tapering until 03/2009, and 09/2008 MTX 10 mg/wk until 02/2010 → CR		
				TCR clonality + in skin lesion (2008), LD marginally elevated 232 U/l (normal range 115–235 U/l) at the time of diagnosis	relapse 05/2010 → MTX 7,5 mg/wk → CR		
2	M27 (case 8 in [5]	Subcutaneous nodules in forehead and scalp (horse shoe shape) in 2005, SPTL diagnosis 12/2006	No B-symptoms	Cervical, thoracic and abdominal CT normal	01/2007 prednisolone	CR since 02/2008	
			Cervical lymph nodes enlarged (since mononucleosis years earlier )	BM normal	60 mg/d (2 weeks) for 3 weeks → CR		
			→ reactive histology	ANA-ab 320 (centromeric staining), ENA-ab positive but specific RNP-, SSA-, SSB- and Sm-abs negative	04/2007 prednisolone		
					80 mg/d until 01/2008 → CR		
3	F66	09/2003 small, reddish papules in upper and lower extremities and nodules on shoulders; histology in 2004: lupus erythematosus profundus; treated with hydroxychloroquiine 300 mg/d (06-12/2005), dapsone 50 mg/d (01-04/2008) with no response	Hypertension, dyslipidemy	Thrombocytosis, leukopenia since 1999 → BM normal, chromosomes normal	05/2008 prednisolone	CR since 03/2011	
			Photosensitivity → photoprovocation negative	→ no specific diagnosis	60 mg/d (2 weeks) decreasing until 12/2008 → CR, relapse 01/2009 – 06/2009 MTX10 mg/wk, prednisolone 40 mg /d (2 weeks) decreasing until 04/2010 → CR, relapsing disease		
				Low CD4 levels in 2008: 0,065 - 0,150/17-23% (normal range 0,458-1,406 E9/l/29-59%) → sulfatrimetoprim prophylaxis	12/2009 – 02/2011 bexarotene 225 mg/d** → initially PR, then PD		
		SPTL diagnosis in 05/2008		No ANA, ENA or DNA antibodies	02/2011 prednisolone 10 mg/d + MTX 5 mg/wk maintenance → CR		
				TCR clonality + in blood (07/2008) and in MB (12/2009), LD slightly elevated 248–380 U/l in 05/2009 – 01/2011			
				CT normal			
4	M 47	Subcutaneous tender nodules on buttock (15 cm) and trunk in 10/2013, SPTL diagnosed in 2/2014	Fatigue, daily fever up to 38,5C, cough, joint pains; no concomitant diseases	WBC 2,6 x 10^9^/l, B-Ly 0,88 x 10^9^/l (33%), B-T-CD4 0,271 x 10^9^/l, CRP 11, total ENA abs 1,6 (ref. <0,7)	Prednisolone 80 mg/d for one week, then 50 mg/d for 1,5 months	CCR after 3 months, all laboratory values normalized (ALT 79), patient returned back to work	
				BM normal, TCR clonality in lymph node, ALP 109, ALT 545, LD 1162 U/l, CT : few 1 cm lymph nodes in right axilla and left inguinal, liver slightly enlarged 16 cm	- > 30 mg/d		
5	F 60 case 7 in [5]	Subcutaneous, firm nodules on fore-head (1 cm) and on upper extremities (blueish) in 2005; SPTL diagnosis in 12/2006	No B-symptoms, no enlarged lymph nodes	CBC normal, LD ad 299 U/l until 06/2009, thereafter normalized	03/2007 EB therapy → PR, 06/2007 prednisolone 60 mg/d (2 weeks) decreasing doses until 11/07 → CR	PR	
			Psoriatic arthropathy treated with leflunomide 20 mg/d 05/2003 – 12/2006	No ANA, ENA or DNA abs, M-component in serum, decreased during follow-up	03/2008 prednisolone		
			Hypertension Dyslipidemy	Thoracic and cervical CT normal	60 mg/d (2 weeks) decreasing doses until 12/2009 → PR		
					12/2008 MTX 7,5 mg – 12,5 mg/wk → stopped 10/2010 → PR		
					10/2010 bexarotene		
					225 mg/d → stopped 02/2011 (ALT elevation 110 U/l) → initially PR then PD		
					05-06/2011 EB therapy → SD, 08-11/2011 CHOP → PR		
6	F39	Subcutaneous, firm nodules in upper and lower extremities, and trunk in 2008; 2/2010 lobular panniculitis in biopsy, 5/2011 SPTL histologically	No B-symptoms, no enlarged lymph nodes; some joint pains concomitantly, hypothyreosis since years	CBCnormal; no ANA, ENA, DNA , TPO or Thygl abs, RF 59 IU/ml (reference 0–14), LD normal; CT normal	8/2011 prednisolone 20– 60 mg/d → CR , relapse in 8/2012, whereafter prednisolone 15 mg/d + MTX 12,5 mg/wk → CR	CR in 1/2013	
7	F14 case 9 in [5]	Subcutaneous nodules in abdominal region, lower and upper extremities in 01/2006, SPTL diagnosis in 02/2007	No B-symptoms, no enlarged lymph nodes	CBC normal at the time of diagnosis, thereafter mild anemia (Hb 110–120 g/l)	02/2007 prednisolone	CR since 09/2009	
			Atopic constitution	No ANA, ENA or DNA antibodies	40 mg/d for 1,5 months → CR (thereafter spontaneously resolving lesions)		
				LD slightly elevated (242) U/l) at the time of diagnosis, thereafter normalized			
				Thorax CT normal			
8	F61	SPTL diagnosis in 03/2001, involvement of both lower legs	Multiple analgetic intolerance	LD slightly elevated 236 U/l	Initial therapy: radiation therapy and CHOP 6 cycles → CR, relapse in 2004, bexarotene and steroids from 2004 → PR/SD	SD	
9	F77	SPTL diagnosis in 09/2007, involvement of extremities and thoracic area	Liver cysts, uterine myoma	LD slightly elevated 259 U/l	Encapsulated doxorubicin 8 cycles + prednisolone → PR	DOD 05/2009	
					IFNα 3 x 6 mio		
					Radiation right thigh 36 Gy		
					Thoracic lesions: triamcinolone intralesional		
14	F68	07/2011, two nodular lesions of the lower limb and a nasal tumor. SPTL confirmed 09/2011	Weight loss, asthenia and fever. Splenomegaly and adenopathies. history of vasculitis between 1994 and 2007	Cytopenia (lymphopenia and thrombocytopenia), elevated liver enzymes, LD >2 N, b2microglobulin 7 mg/L	Chemotherapy with etoposide (08/2011), followed by CHEP (09/2011-10/2011) and CHOP (11/2011-02/2012).	CR since 03/2012.	
15	M*** [6]	In 02/2007, three plaques on the left and right upper limb (5-10 cm), and face; diagnosis confirmed in 06/2007	Fever, asthenia, hepatomegaly and splenomegaly, macrophage activation syndrome	LD >2 N, elevated liver enzymes, ANA and anti SSA +, TCR clonality + .	Corticosteroid and cyclosporine A (2007–2010)	CR in 03/2008 without relapse.	
16	F22	Medical history of cytophagic and histiocytic panniculitis in 1993, treated with corticosteroid. SPTL diagnosed in 2000, widespread plaque and nodule lesions on upper and lower limbs, trunk and face.	Fever, weight loss, asthenia, hepatomegaly and splenomegaly. Macrophage activation syndrome.	LD >2 N. No ANA abs, b2microglobulin >7 mg/L	Chemotherapy with autologous stem cell transplantation in 2001, relapse in 2001. Corticosteroid and MTX between 2002 and 2008 - > CR	CR in 11/2002, no relapse.	
17	F37	EN in 2010. In 01/2011, 8 nodules on trunk and face. SPTL diagnosed in 04/2011.	Asthenia, myalgia and diarrhea.	Lymphopenia, No ANA abs, TCR clonality +	Hydroxychloroquine in 07/2011	CR in 10/2011, no relapse.	
18	M48	AL amyloidosis in 07/2007 (chemotherapy + autologous stem cell transplantation). In 04/2011, 3 nodules (lower limb,trunk). SPTL confirmed in 09/2011	Adenopathies, hepatomegaly and splenomegaly	Leukopenia, anemia, thrombocytopenia, TCR clonality +	Cyclophosphamide, adriamycin, vincristine and methylprednisolone in 09/2011.	PR, deceased in 04/2012 (infectious pneumopathy)	
19	F50	Multiple nodules of the upper and lower limbs and trunk in 01/2011. SPTL confirmed in 02/2011	-	Elevated liver enzyme and LD.	Corticosteroid in 04/2011	CR in 07/2011	
20	F15	One isolated nodule (>10 cm) of the lower limb in 12/2011. SPTL confirmed in 04/2012	Fever, asthenia, weight loss, adenopathy	Leucopenia, lymphopenia, elevated LDH. TCR clonality +	Corticosteroid (05-06/2012), vinblastine 05/2012), multiple courses of chemotherapy since 06/2012	PR in 01/2013	

(Clinical details of cases 10–13, all in remission, are reported in [5], in Table 1 as cases 3–6).

=*at the time of diagnosis.

**Since Mehta and coworkers recently reported promising results of bexarotene therapy in SPTL, [7], bexarotene was used for the treatment of three of our patients: cases 3, 5 and 7. Only case 7 remained in long-lasting remission with a combination therapy of bexarotene and steroids, and cases 3 and 5 reached only a temporary initial partial response [5].

***Case 15 reported first in [6] at the age of 9 months.

CT = computed tomography, LD = lactate dehydrogenase, mtx = methotrexate, CR = complete remission, PR = partial remission, MF = mycosis fungoides, ANA = antinuclear antibody, ENA = extractable nuclear antigen, BM = bone marrow, PD = progressing disease, SD = stable disease, CBC = complete blood count, WBC = white blood cells, RBC = red blood cells, ALP = alkaline phosphatase, ALT = alanine aminotransferase, EB = electron beam radiation therapy, CHOP = cyclophosphamide, doxorubisine, oncovine, prednisolone, IFN = interferon, DOD = died of disease.

For the microarray analysis, fresh subcutaneous tissue samples were successfully obtained from four patients with newly diagnosed SPTL (cases 1–4, Table 1). The first samples were taken at the time of diagnosis, before any treatment (pre-treatment), together with a sample for TCR (T-cell receptor) rearrangement analysis (http://www.hus.fi/sairaanhoito/laboratoriot/Sivut/default.aspx). The follow-up samples were first obtained three to six months after the start of systemic treatment (treatment1) and second follow-up samples six to 12 months after the initiation of the therapy (treatment2) when a complete clinical response was reached (biopsy from the region of pre-existing lesions). In addition, one treatment1 sample was obtained from a patient (case 5, Table 1) with no matching pre-treatment or treatment2 sample. The control RNA for microarray studies consisted of two samples from normal subcutaneous fat tissue (FAT1 and −2, derived from patients undergoing dermatologic surgery) and two cases of non-malignant panniculitis, erythema nodosum (EN1 and −2). The study was approved by the Medical Ethical Review Board of Helsinki University Central Hospital.

For the confirmatory immunohistological studies six cases of lupus erythematosus profundus (LEP, *i.e*. lupus panniculitis), and 13 cases of EN were included. All LEP controls were female (mean age 38.3 years) and two (=33%) of them had a pre-existing collagenosis with immunosuppressive therapy (steroids and hydroxychloroquine). Eleven of 13 (85%) EN cases were female (mean age 37.9 years). In seven (54%) cases the etiology of EN was an infection (*Yersinia* in 67% of them), in two (15%) EN was the first symptom of a systemic disease (colitis ulcerosa and sarcoidosis), and in four, the etiology remained uncertain regardless of thorough examinations. All of the samples were taken prior to immunosuppressive treatment (except for the two LEP cases with a pre-existing collagenosis).

### RNA extraction

The fresh, skin biopsies were immediately immersed in RNALater™ (RNA Stabilization Reagent, Qiagen, Valencia, CA) and stored at −20/70°C. The subcutis and the deeper dermis of the skin biopsy were dissected for the RNA isolation, which was performed with RNeasy Mini Kit/RNeasy Lipid Tissue Mini Kit (Qiagen) according to manufacturers’ instructions. In SPTL samples, the amount of malignant T-cells was over 50% of the mononuclear cell infiltrate, based on cytomorphology in histopathological analysis.

### Hybridization to affymetrix exon array

The RNA used was intact and of high quality (RIN 8.0-10), as confirmed with Agilent 2100 Bioanalyzer at the Biomedicum Helsinki Functional Genomics Unit (FuGU, http://www.helsinki.fi/fugu/*).* The gene expression arrays (Human Exon 1.0ST, Affymetrix) were performed at FuGU according to manufacturer’s instructions. Microarray data are available in the ArrayExpress database [www.ebi.ac.uk/arrayexpress] under accession number E-MTAB-910. (Username: Reviewer_E-MTAB-910, Password: wiknooqq, Experiment E-MTAB-910).

### Analysis of the microarray data

Data from microarrays were pre-processed using background correction and quantile normalization [8]. For each gene, probe set intensities were summarized to obtain a single expression value. Differential expression analysis was done for the following comparisons: (1) pre-treatment SPTL (n = 4) vs. normal subcutaneous fat tissue (n = 2), (2) pre-treatment SPTL (n = 4) vs. EN (n = 2), and (3) pre-treatment SPTL (n = 4) vs. combined controls (aforementioned normal subcutaneous fat tissue and EN; n = 4). In each comparison, genes with median fold change (FC) >4 (<0.25) and p-value of t-test <0.05 were considered as differentially expressed. Data analysis was done using the Anduril bioinformatics framework [9]. Due to the small number of samples available for this rare disease, false discovery correction for p-values was not used. Rather, the key findings were confirmed with qRT-PCR and immunohistochemistry.

### Relative quantification of gene expression

We confirmed the expression of three relevant genes, *CXCR3, IDO-1,* and *IFNG* by quantitative RT-PCR. The SPTL RNA samples (n = 5) were extracted either from fresh RNA later stabilized skin tissues (cases 2–3 in Table 1 used also in the arrays) or from formalin-fixed-paraffin-embedded (FFPE) skin tissues (cases 1, 5–6 in Table 1, NucleoSpin FFPE RNA 740969.10 Macherey-Nagel GmbH, Germany) according to manufacturer’s instructions. Three EN RNA samples were used in qRT-PCR as reference tissue. Two samples were the same used in the arrays (EN1 and EN2) and the third was extracted from the FFPE sample. The reverse transcription into cDNA was performed using SuperScript® VILO cDNA Synthesis kit (11754–050, Invitrogen). We used the following Taqman Assays (*IDO-1*; Hs00984148_m1, 66 bp n = 5, *IFNG*; Hs00989291_m1, 73 bp n = 4, *CXCR3*; Hs00171041_m1, 111 bp n = 4) and iQ Supermix (170–8860, Bio-Rad) and LightCycler 1.5 System (Roche) for the amplifications. The size and the purity of the amplicons were checked with agarose gel electrophoresis (2,5% SeaKem® LE agarose, Rockland, ME USA 1xTBE). The relative expression levels were normalized to reference gene *GAPDH* (Taqman assay 4310884E, 118 bp) and further compared to expression levels in reference tissue, erythema nodosum according to 2^-∆∆CP^-method [10].

### Immunohistochemistry and confocal microscopy

As part of routine diagnostics at the Dermatopathology laboratory of Helsinki University Central Hospital, all tissue samples were immunostained for the following markers (manufacturer and dilutions given in parenthesis): CD3 (Novocastra, New Castle, UK; 1:100), CD4 (Novocastra; 1:150), CD5 (Novocastra; 1:25), CD7 (Novocastra; 1:100), CD8 (Novocastra; 1:25), CD30 (Dako, Glostrup, Denmark; 1:25), CD56 (Zymed, South San Fransisco, CA, USA; 1:50), GZMB (Monosan, Uden, The Netherlands; 1:100), TIA1 (Biocare, Birmingham, UK; 1:200), Ki-67 (MIB-I antibody, Dako, Glostrup, Denmark; 1:50), and TCR alpha/beta (GeneTex, TX, USA; 1:100) according to the manufacturers’ instructions, and visualized with DakoEnvision (Glostrup, Denmark).

Additionally, immunohistochemical (IHC) detection of the following proteins CXCL9 (Abcam, Cambridge, UK; 1:500), IL2RB (Abcam, 1:200), IDO-1 (Chemicon International Inc. USA; 1:100, clone MAB5412), FoxP3 (SpringBioscience; 1:50 clone SP97), and CXCR3 (Abcam, 1:500), was performed according to the manufacturers’ instructions and ImmPRESS Universal Antibody (anti-mouse Ig/anti-rabbit Ig, peroxidase) Polymer Detection Kit (Vector Laboratories, Burlingame, California) and NovaRED (Vector Laboratories, Burlingame, CA) or AEC (Abcam) chromogens. Furthermore, double IHC stainings were performed for CD8 (1:100)/IDO-1 (1:100), and also for CD68 (Spring Bioscience, Pleasanton, CA, USA, 1:200)/IDO-1 (1:100) according to the manufacturers’ instructions and using Vector Elite PK-6101 Rabbit IgG (Vector Laboraties)/Permanent HRP Green Kit KDB10049 (Nordic BioSite AB, Täby, Sweden) and VECTASTAIN AP Mouse IgG Kit(Vector Laboratories, AK-5002)/Permanent AP-Red Kit, BCB20041 (Biosite), respectively. Double IHC staining for CD8/CXCR3 was performed using MACH2 Double Stain 2 Mouse-HRP + Rabbit-AP Polymer Detection Kit (cat.nro 901-MRCT525-021709, Biocare Medical, Concord California) with BCIP/NBT and AEC as chromogens, respectively. Moreover, double immunofluorescence (IF) staining for CD11c (Bio SB, Santa Barbara, CA, 1:50)/IDO-1(1:100) was performed according to the manufacturers’ instructions and using AlexaFluor-594 anti-rabbit antibody (red, Abcam, 1:1000) and AlexaFluor-488 anti-mouse antibody (green, Abcam 1:1000), respectively. Immunofluorescence stainings were analyzed and photographed using Leica Confocal Microscopy (Leica Microsystems). The IHC detection was carried out on the total of 42 FFPE tissue samples, obtained from 20 SPTL patients (23 samples), six cases with LEP and 13 cases of EN. For each IHC, several technical controls were included for both positive and negative reactions. The positive staining result was graded as follows: − indicates <10%, +10-25%, ++ 25-50%, and +++ over 50% of the lymphocytes expressed the given marker.

## Results

### SPTL skin samples demonstrate up regulation of *IDO-1* and Th1 type cytokines

We first compared SPTL skin samples to normal subcutaneous fat tissue to exclude the effect of normal fat gene expression. We identified altogether 968 genes differentially expressed in untreated/pre-treatment SPTL skin samples, out of which 589 were up regulated and 379 were down regulated. The three most strikingly overexpressed genes in the SPTL lesions were chemokine (C-X-C motif) ligand 10 (*CXCL10;* fold change: 171), guanylate binding protein 5 (*GBP5;* FC: 78), and indoleamine 2,3-dioxygenase (*IDO-1*; FC: 71). Likewise, the expression levels of *CXCL11* (FC: 41) and *CXCR3* (FC: 10), were elevated. Moreover, interleukin 2 receptor β (*IL2RB*; FC: 46), chemokine (C-C motif) ligand 5 (*CCL5 = RANTES*; FC 35), and interferon gamma (*IFNG*; FC 17) were highly expressed. The data also shows the overexpression of perforin 1 (*PRF1;* FC 33), different granzymes (e.g. *GZMA;* FC 25, *GZMB*; FC 23), and members of the SLAM family *(SLAMF1;* FC 6, *SLAMF6*; FC 36, *SLAMF7*; FC 29, and *SLAMF8*; FC 23). Interestingly, of the TRIM family genes (TRIM59; FC 10 and TRIM14; FC 6) were upregulated in this comparison (Table 2).

**Table 2:** **SPTL patients show differences in the expression of selected genes compared to controls**

**Gene**	**Normal fat** ^**1**^		**Combined controls** ^**2**^		**Inflammatory EN** ^**3**^		**DNA band**	**Gene function**
	**FC**	**p-value**	**FC**	**p-value**	**FC**	**p-value**		
*CXCL10*	170.1	<0.01	32.93	0.05	ns	ns	4q21.1	chemokine, T-cell trafficking, ligand for CXCR3
*GBP5*	78.08	<0.01	28.00	0.02	ns	ns	1p22.2	GTPase activity, cellular response to interferon-G
*IDO1*	70.57	<0.01	35.52	<0.01	17.91	0.01	8p12-11	catabolism of tryptophan, suppressor of immune response
*IKZF3*	63.27	0.05	24.48	0.01	10.98	0.05	17q12	B-cell activation, regulation of lymphocyte differentiation
*IGJ*	55.37	0.01	36.09	0.01	19.40	0.03	4q13.3	IgA and antigen binding, adaptive immune response,
*CXCL9*	ns	ns	20.25	0.04	ns	ns	4q21.1	chemokine, T-cell trafficking, ligand for CXCR3
*IL2RB*	45.71	<0.01	25.76	<0.01	12.49	0.01	22q12.3	cytokine receptor
*CXCL11*	41.41	<0.01	17.31	0.01	7.33	0.02	4q21.1	chemokine, T-cell trafficking, ligand for CXCR3
*KLRD1*	36.53	0.01	28.68	0.01	19.44	0.01	12p13.2	transmembrane receptor activity, innate immune response
*CCL5*	34.83	<0.01	18.37	0.01	ns	ns	17q12	chemokine, T-cell polarization
*PRF1*	32.69	<0.01	26.15	<0.01	19.86	<0.01	10q22.1	calcium ion binding, cellular defense response,
*SLAMF7*	28,67	0.01	16.60	<0.01	9.89	0.01	1q23.3	lymphocyte activation
*GZMB*	22.58	<0.01	22.58	<0.01	18.57	<0.01	14q12	T-cell cytotoxicity
*CCR5*	25.72	<0.01	10.21	0.01	4.75	<0.01	3p21.31	chemokine receptor
*NKG7*	23.55	<0.01	17.73	<0.01	13.82	<0.01	19q13.41	integral component of plasma membrane
*RASGRP1*	20.76	0.03	8.09	0.01	4.10	<0.01	15q14	lymphocyte regulation
*APOBEC3G*	19.47	<0.01	9.65	<0.01	5.42	<0.01	22q13.1	innate immune response, defense response to virus
*KIR2DS4*	ns	ns	ns	ns	19,01	0.05	19q13.42	innate immune response
*IFNG*	17.24	0.01	15.46	0.01	10.45	0.01	12q15	cytokine, immunoregulator
*TNFRSF9*	15.54	0.02	14.45	0.02	11.57	0.02	1p36.23	receptor, survival and development of T cells
*CCL4*	12.64	<0.01	8.97	0.01	ns	ns	17q12	chemokine, T-cell polarization
*TRIM59*	9.93	0.01	5.38	0.03	ns	ns	3q25.33	negative regulation of I-kappaB kinase/NF-kappaB signaling
*CXCR3*	9.83	0.04	6.68	<0.01	4.68	0.04	Xq13.1	chemokine receptor, recruitment of inflammatory cells
*FASLG*	5.00	0.01	5.38	<0.01	6.12	0.01	1q24.3	cytokine activity, T cell apoptotic process
*TBX18*	0.22	0.05	ns	ns	ns	ns	6q14.3	transcription factor
*TBX15*	0.13	<0.01	0.19	<0.01	ns	ns	1p12	transcription factor

^1^SPTL vs. normal subcutaneous fat, ^2^SPTL vs. combined controls, ^3^SPTL vs. inflammatory EN, ns = non-significant. FC = Fold Change. See ArrayExpress for all data.

**Figure 2:**
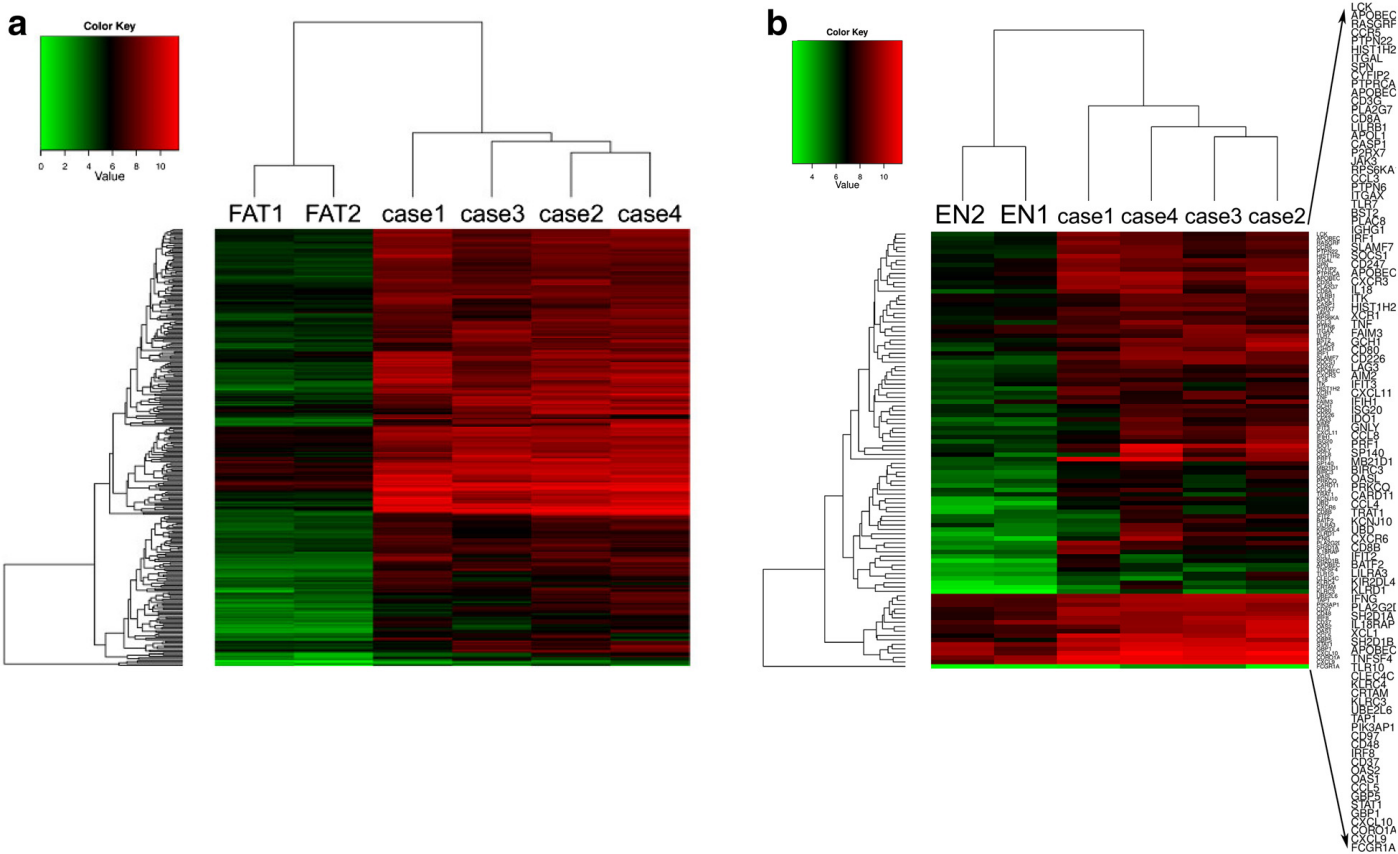
**Expression profiling revealed a gene expression pattern distinguishing SPTL from combined control samples. a)** Visualization of 290 genes that are overexpressed between pre-treatment (n = 4) and combined control samples (n = 4). Only the normal fat control samples are shown. Expression values are in base-2 logarithm scale. **b)** Visualization of 99 genes from set a) above, annotated with the Gene Ontology term “Defence response” (GO:0006952). Here, the erythema nodosum (EN) control samples with a similar type of tissue inflammation, but devoid of malignant T lymphocytes, are shown in turn.

We then compared the pre-treatment SPTL samples with *in silico* “combined controls” of normal subcutaneous fat tissue and non-malignant panniculitis, erythema nodosum (EN) samples, *i.e*. the median values yielded by the latter two groups. A visualization of 290 genes that are overexpressed between SPTL samples and combined controls is presented as a heatmap (Figure 2a). In this comparison, the aforementioned genes stayed overexpressed, but to a slightly weaker extent (Table 2). Three most overexpressed genes were *IGJ, IDO-1*, and *CXCL10* (Table 2). To note, the overexpression of *CXCL9* (FC 20) was seen only in this combined comparison (Table 2). Furthermore, the 99 genes that are annotated with the Gene Ontology term “Defence response” are visualized in a heatmap as well (Figure 2b).

When comparing SPTL samples against inflammatory EN samples only, six genes (*PRF1, KLRD1, IGJ, KIR2DS4, GZMB*, and *IDO-1*) showed the highest expression and appeared equally overexpressed by 20-fold. Also, *IFNG, IL2RB*, and CXCR3 stayed overexpressed but to a slightly weaker tendency as observed earlier (Table 2). Of the CXCR3 ligands, only CXCL11 reached significant overexpression in this comparison (Table 2). This would suggest some similarity between SPTL and EN regarding the CXCR3 pathway involved in the development of autoimmune diseases (reviewed by [11]).

Consistent with previous findings, the expression of *GZMB* gene encoding the cytotoxic protein GZMB, used in the routine diagnostics of SPTL, was constantly approximately 20-fold overexpressed in all comparisons. Likewise, Fas ligand (*FASLG*, TNF superfamily, member 6) was equally overexpressed by 5-fold in all comparisons. In addition, up regulation of *RASGRP1*, a nucleotide exchange factor specifically activating Ras, was observed in SPTL samples (FC 21, FC 8, and FC 4). From the NK gene family, only *NKG7* (natural killer cell group 7 sequence) was overexpressed by 23-, 18-, and 14-fold, respectively. Other up regulated genes in this comparison remained in line with the other comparisons. Of the down regulated genes, several T-box transcription factors (*TBX18, TBX15*) were represented. Among the miRNA family (*hsa-miR-199a-2, hsa-miR-410, hsa-miR-487-b, and hsa-miR-3665)* were down regulated by 5-10-fold, respectively. On the other hand, *miR-219-1* was overexpressed (FC 4). The list of discussed, deregulated genes is summarized in Table 2.

In the set of the additional follow-up samples, obtained during the systemic therapy with prednisolone and low-dose methotrexate, a clear transition towards normalization of the most relevant genes, like *IDO-1*, was observed as early signs of response at the time when the malignant T cell population was still clearly detectable histologically (Additional file 1: Figure S1).

### Relative quantification of gene expression

**Figure 3:**
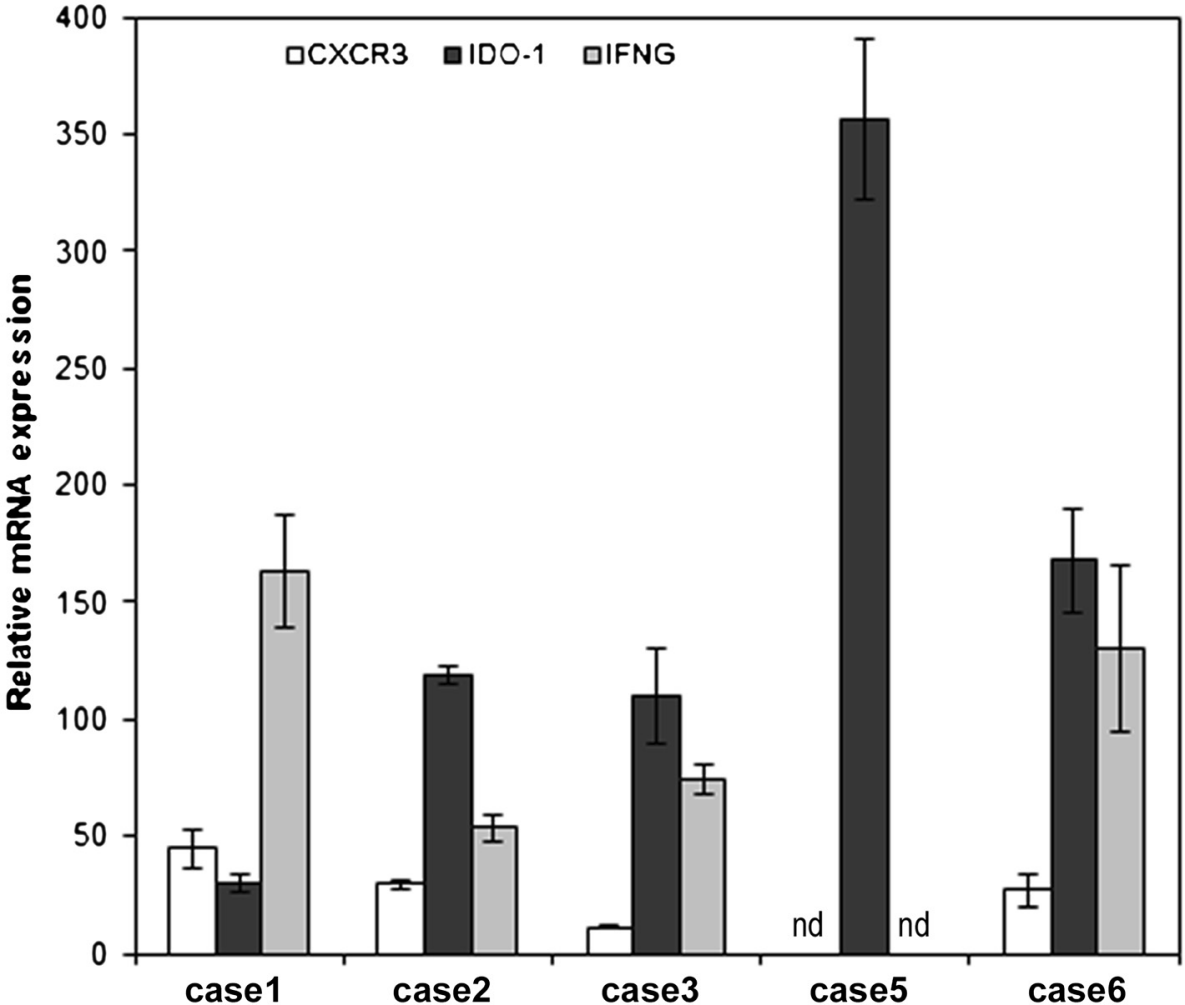
**Relative quantification of studied gene expressions in SPTL lesions compared with reference tissue (erythema nodosum).** Relative mRNA expression of *CXCR3* (white column), *IDO-1* (dark grey), and *IFNG* (light grey) in SPTL lesions. Relative expressions are represented as fold changes in comparison with erythema nodosum*.* To note, *CXCR3* and *IFNG* genes showed no detectable expression in case 5, although the *GAPDH* level was similar to other samples. All gene expression levels were normalized to reference gene, *GAPDH*. nd = not detected.

The expression levels of three selected deregulated genes, *CXCR3*, *IDO-1,* and *IFNG* were confirmed by quantitative RT-PCR from five cases and normalized to reference gene, *GAPDH*. The relative mRNA expression levels were then compared with the levels in reference tissue (erythema nodosum, EN) and results were presented as fold changes. *IDO-1* mRNA showed overexpression by 30-350-fold in all SPTL samples compared with EN (Figure 3). The cytokine receptor *CXCR3* showed overexpression by 10-50-fold and *IFNG* by 50-150-fold compared to EN. We did not detect any CXCR3 or IFNG expression in case 5, although the GAPDH levels were similar to other samples. Overall, the quantitative analysis not only confirmed the expression of *IDO-1* and *CXCR3* but also revealed even higher fold changes than the microarray analyses.

### Immunohistochemistry designates the cellular origin of the deregulated gene products

**Figure 4:**
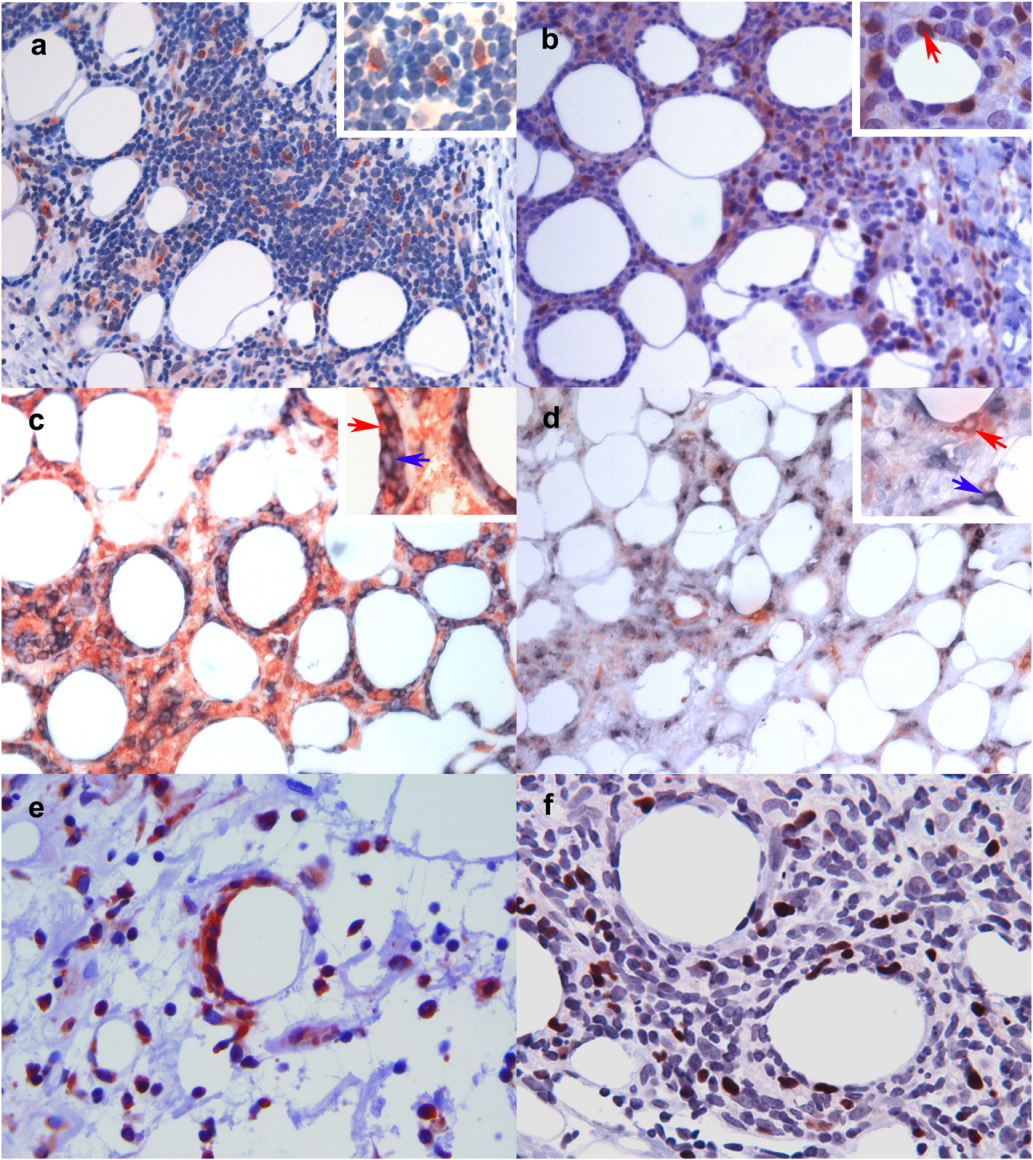
**Immunohistological confirmation of the protein expression of the up regulated genes in SPTL. a)** CXCL9-expressing, morphologically mostly malignant lymphocytes in a SPTL lesion (red, 20x). **b)** IDO-1-expressing morphologically malignant lymphocytes (red arrow) rimming a fat cell in a SPTL lesion (red, 20x). **c)** Double immunostaining for CD8 (cyan) and CXCR3 (red) showing CD8^+^CXCR3^+^ lymphocytes (red arrow) in a SPTL lesion (20x). Cells expressing only CD8 are indicated with blue arrow. No counterstain. **d)** Double immunostaining for CD8 (cyan) and CXCR3 (red) showing exclusively the expression of CD8 and CXCR3 in different cells in a LEP lesion (20x). No counterstain. a)-d) Insert in upper right corner represents magnification of 40x. **e)** CXCR3-expressing malignant lymphocytes rimming the fat cell in a SPTL lesion (red, 20x). **f)** High number of FoxP3+ (brown) regulatory T-cells in a SPTL lesion (red, 40x).

**Figure 5:**
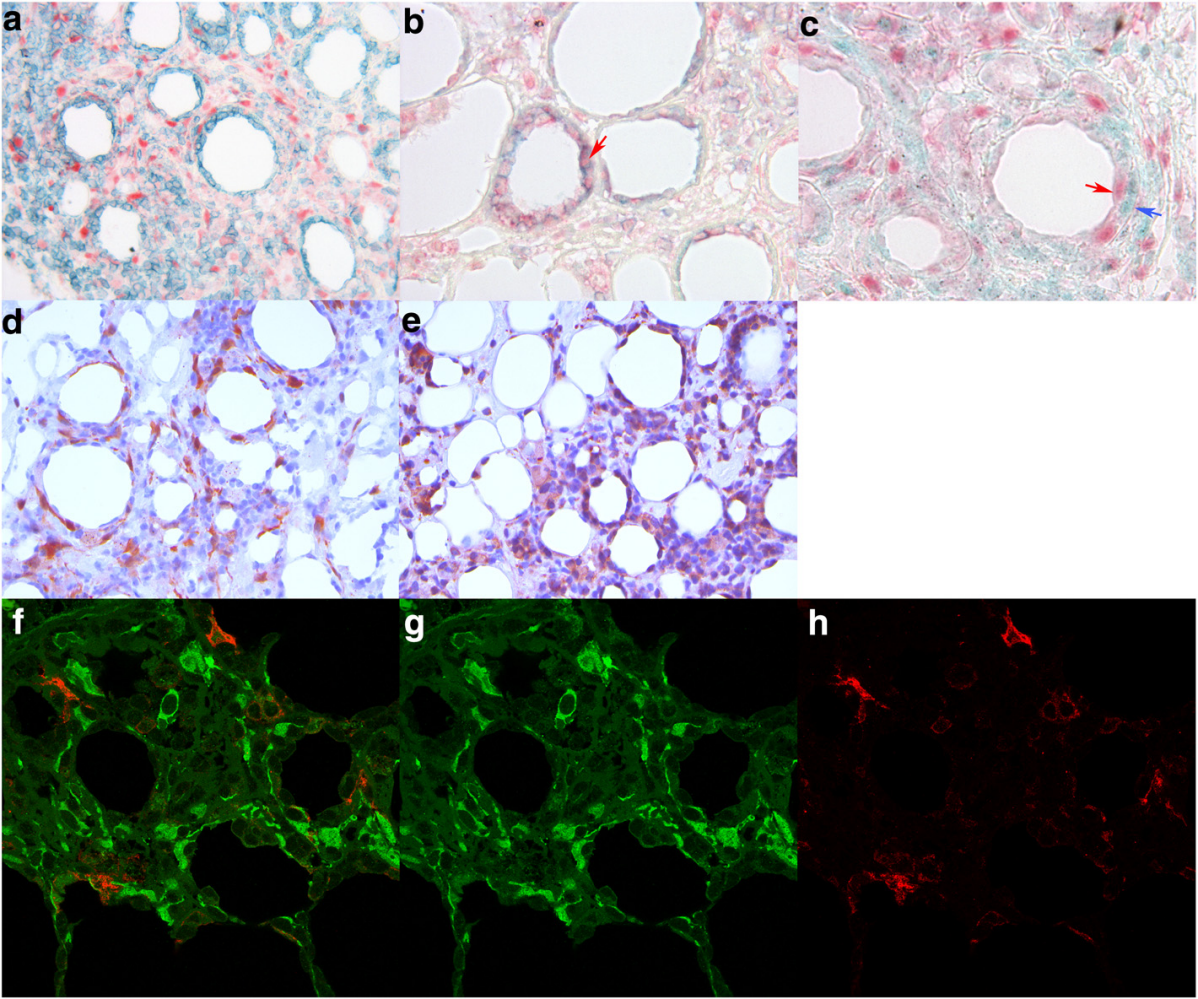
**Immunohistological specification of the immunosuppression-inducing IDO-1 in SPTL. a)** Double IHC staining of CD8 (cyan) and IDO-1 (red) shows that IDO-1 is mostly expressed in other cells than CD8+ lymphocytes in a SPTL lesion (20x). **b)** Here, double staining of CD8 (cyan) and CXCR3 (red) shows the expressions in same cells in SPTL as comparison (red arrow, 40x). **c)** Double staining of IDO-1 (red, red arrow) and the macrophage marker CD68 (cyan, blue arrow) shows expression mainly in different cells (40x). **d)**-**e)** Perinodular fat, infiltrated with morphologically malignant lymphocytes, surrounding an enlarged lymph node of a SPTL patient (case 4, Table 1). Similar **d)** IDO-1 and **e)** CXCR3 expression is seen as in subcutaneous SPTL lesions. **f)**-**h)** Double IF staining of CD11c (red) and IDO-1 (green) confirms that IDO-1 is not expressed by CD11c -positive dendritic cells (Leica confocal microscopy, 40x).

To further confirm the cellular origin of the deregulated gene products, we performed IHC of selected gene products in an extended series of patient and control tissue samples. IDO-1 was seen intensively expressed both in some morphologically malignant T-cells rimming the fat cells (Table 3, Figures 4b and 5c) as well as in nearby CD8^−^CD68^−^CD11c^−^ cells (Figures 5a,c,f) [12,13], when using double immunofluorescence. Surprisingly, IDO-1 expression was not detected in CD11c + dendritic cells with confocal microscopy performed on double-IF stained sections (Figures 5f-h). We also searched for FoxP3-positive regulatory T-cells (Tregs), since IDO-1 has been reported to increase the proportion of Tregs in the tumor infiltrate [14]. Approximately 25-50% of the inflammatory lymphocytes were FoxP3-positive in SPTL samples (Figure 4f) with abundant expression of IDO-1 (Table 3). FoxP3^+^ lymphocytes were found also within the inflammatory infiltrates of EN and LEP, both with no IDO-1 expression, but at a considerably lower frequency (Table 3).

**Table 3:** **Protein expression of selected up regulated genes as detected by immunohistochemistry in tissue sections of SPTL, lupus erythematosus panniculitis (LEP) and erythema nodosum (EN)**

**Diagnosis**	**Positivity among**	**IDO-1**	**CXCR3**	**CXCL9**	**IL2RB**	**FOXP3**
SPTL	morphologically malignant cells in adipose tissue	14/19	15/21	15/15	9/14	0/9
		++	++	+ + +	+ +	-
	inflammatory infiltrate in dermis	11/19	2/21	4/14	9/14	7/9
		++	+	+	+	+ or ++
Lupus erythematosus panniculitis (LEP)	inflammatory infiltrate	0/5	5/5	6/6	4/6	4/5
		-	+	+ +	+	+
Erythema nodosum (EN)	inflammatory infiltrate	0/5	11/12	9/10	8/9	1/5
		-	+ + +	+	+	+

Number of positive cases/all cases studied.

Grading of positive immunostaining: − below 10%, +10-25%, ++ 25-50%, and +++ over 50% of the cells (lymphocytes) expressing the given marker. To note, the positive cells were not always equally distributed in the tissue sections. LEP and EN have no morphologically malignant cells.

CXCL9 and CXCR3 were chosen as markers for CXCR3 pathway. CXCR3 protein was expressed almost exclusively in the malignant lymphocyte infiltrates of the SPTL samples (Table 3, Figures 4c,e, 5b). The non-malignant inflammatory infiltrates of LEP and EN control samples expressed CXCR3 protein, too, but to a varying extent. (Table 3, Figure 4d). CXCL9 was abundantly expressed in the malignant cells of 15 SPTL samples (Figure 4a, Table 3) while notably less so in all LEP and EN samples (Table 3). By double immunostaining of SPTL samples for CD8 and CXCR3, we confirmed that the malignant lymphocytes, typically rimming the adipocytes, mostly co-expressed both markers (Figures 4c and 5b). In LEP, the CXCR3 and CD8 were not co-expressed by the same cells (Figure 4d).

Interestingly, we could also examine the biopsies of affected lymph nodes of one of the SPTL patients (case 4, Table 1). An intense malignant T-cell infiltration, rimming the fat cells in the adipose tissue surrounding the nodes, was found. The pattern of IDO-1 and CXCR3 expression was similar to that seen in the cutaneous SPTL lesions (Figure 5d-e), as well as FoxP3 expression (data not shown). To conclude, markers of CXCR3 pathway, typically involved in autoimmune diseases, were expressed both in SPTL and in control (LEP and EN) cases, but in SPTL, the main source of CXCL9 and CXCR3-positive cells was the malignant, CD8+ lymphocyte infiltrate (Table 3).

## Discussion

This is the first study to explore the gene expression signatures relevant for the pathogenesis of SPTL. Obtaining fresh, lesional tissue samples of this rare lymphoma has been challenging and therefore, the findings were validated in a larger archival material of 23 SPTL samples, with several confirmatory methods and comparisons such as qRT-PCR and/or single and combined IHC.

The most important and novel finding is that SPTL lesions are characterized by high expression of the immunosuppressive protein IDO-1 (Indoleamine 2,3-dioxygenase), both by the morphologically malignant T cells in addition to CD11c-CD68- double negative cells in the microenvironment. This result is supported by the high levels of various inflammatory cytokines shown here, e.g. IFNG, which is also known to induce the expression of IDO-1. Furthermore, IDO-1 is an essential enzyme in tryptophan catabolism, along the kynurenin pathway [15]. IDO has been identified as a key regulator of tumor immune evasion. IDO-1 has been shown to protect tumors from an attack by tumor-associated, antigen-specific host cytotoxic T cells [16]. Increased IDO expression and activity has been reported in many malignant diseases, including hematological malignancies [17], but never earlier in SPTL. The expression of IDO-1 also seems to decrease the infiltration of immune cells in the tumor and increase the proportion of regulatory T lymphocytes (Tregs) in the infiltrate [14]. We found the expression of FoxP3+ Tregs to associate with the IDO-1 expression in SPTL.

The relationship between IDO-1-expressing tumors and T lymphocytes is, however, complex as IFNG is a major inducer of IDO-1 [15,18]. IFNG is a soluble cytokine, predominantly produced by the NK cells with antiviral, immunoregulatory, and anti-tumor properties. Its aberrant expression is associated with several autoimmune diseases [19]. In our series, *IFNG* was 17-fold up regulated in SPTL samples, and its expression lowered to 4.5-fold as a consequence of ongoing therapy (Additional file 1: Figure S1). IFNG has been shown to act in a feedback fashion to induce IDO-1′s enzymatic function. This then creates an immunosuppressive microenvironment, through the immunosuppressive kynurenin metabolites, leading to T cell anergy towards the transformed tumor cells [20,21], reviewed in [14,22].

We also found a distinct expression pattern intensifying a Th1 type response through high expression of *CXCR3* and *CCR5*, the Th1 receptors, and their ligands *(CXCL9, CXCL10, CXCL11, CCL5,* and *CCL4)* in SPTL. CXCR3 is expressed on several immune cells, mainly on natural killer cells and activated T helper cells polarized into Th1 direction but also on subset(s) of circulating human Tregs [23]. The CXCR3 ligands CXCL9, CXCL10, and CXCL11 are members of the CXC chemokine family, not constitutively expressed but also up regulated by *e.g.* IFNG in a proinflammatory cytokine milieu. They are expressed by T lymphocytes (CD3, CD4, and CD8 populations), and moreover, CXCL9 and CXCL10 are chemo attractants for CD4+ and CD8+ T effector cells [11]. In cutaneous LE, CXCR3 is expressed by a majority of the infiltrating T cells, and the three CXCR3-activating chemokines are produced locally [24]. The role of CXCR3 pathway has been proven central in the development of many autoimmune diseases, such as rheumatoid arthritis, SLE and autoimmune thyroid diseases [25,26], reviewed by [11]. In this study, although *CXCL9* and *CXCR3* were expressed – to a varying degree – also in the inflammatory dermatoses LEP and EN, they were mostly expressed by the malignant lymphocytes in SPTL as shown by immunohistochemistry. With double IHC we further showed that the CD8^+^- malignant cells rimming the adipocytes also expressed CXCR3.

*IFNG* overexpression has been reported in cutaneous LE subtypes [27] and other autoimmune diseases (reviewed in [28]). It is thus not surprising that IFNG- induced IDO-1 has also been shown to drive autoimmunity [29]. Even a specific *IDO-1* gene SNP associates with autoimmune systemic sclerosis [30]. Thus, taken together, the above discussed gene activation observed in this study strongly suggests an autoimmune background for SPTL.

Our data also revealed other interesting genes to be overexpressed in SPTL and related to autoimmunity. The second most up regulated gene was *GBP5* which is known to promote NLRP3 inflammasome assembly and immunity in mammals [31]. *RASGRP1*, is a nucleotide exchange factor specifically activating the Ras pathway. It regulates the threshold of T cell activation and antigen induced expansion and controls the initiation and duration of CD8 T cell immune responses but also activates NK cell effector functions [32]. Additionally, up regulation of CCL5 (known as RANTES), a chemokine mediating the trafficking and homing of T cells [33], in the SPTL samples refers to a Th1 type inflammatory response [34]. Other genes, favoring Th1 type autoimmunity, like *NKG7* [34,35], *IL32* [36], *IL1*8 [37], *CCR1* [38], and *CCR5* [39] were also overexpressed in SPTL based on this array data. Interestingly, in the set of follow-up samples, obtained during the systemic therapy with prednisolone and low-dose methotrexate, the gene expression profile of the most relevant genes, like IDO-1, clearly normalized along the diminished malignant T cell population in the tissue.

It was recently shown in a humanized mouse model that IDO-expressing humanized mesenchymal stem cells (MSC-IDO) were capable of suppressing T-lymphocyte proliferation and promoting tumor growth in melanoma and lymphoma tumor models [40]. Importantly, this effect was reversed by the IDO inhibitor 1-methyl-tryptophan. Thus, our finding of significantly elevated IDO-1 expression in SPTL compared to EN and LEP, is essentially important and of clinical relevance since novel specific IDO inhibitors [41] are already in early clinical testing.

## Conclusions

Our findings indicate that an autoimmune type of inflammation is likely to underlie the development of SPTL and thus corroborate previous speculations on eventual overlap between SPTL and lupus erythematosus [1,3,5]. We hypothesize that some – as yet unknown - triggering factor induces an autoinflammatory reaction with the up regulation of IFNG, CXCR3, and CCL5. This in turn, leads to the up regulation of IFNG-inducible IDO-1, which is known to induce an immunosuppressive microenvironment, allowing malignant cells to escape of immunological control. This is the first study on gene and protein signature in SPTL and it provides a relevant molecular basis for further studies in defining novel targets for future therapeutic efforts.

### Availability of the supporting data

Microarray data are available in the ArrayExpress database [www.ebi.ac.uk/arrayexpress] under accession number E-MTAB-910 (see Methods for more details).

## Additional file

Additional file 1:
**SPTL gene expression profile changes during treatment.** The SPTL skin lesions show changes in the expression of up regulated genes during ongoing clinical response to systemic steroid (40-60 mg/d) and methotrexate (10 mg/week). The bars are presented on logarithmic scale indicating the microarray fold changes of SPTL samples compared to normal subcutaneous fat tissue (n=2). Pre-treatment, treatment1, and -2 values are based on three, three, and two patients, respectively. The gray horizontal line indicates neutral expression level with fold change of one. Statistical significance between SPTL samples and normal subcutaneous fat tissue is indicated with ** (p<0.01), * (p<0.05) and “.” (p<0.1).

## Abbreviations

SPTL: Subcutaneous panniculitis-like T cell lymphoma
EN: Erythema nodosum
SLE: Systemic lupus erythematosus
LEP: Lupus erythematosus panniculitis
TCR: T cell rearrangement
APC: Antigen presenting cell
NK: Natural killer
SNP: Small nucleotide polymorphism
