# Supplementary figures and images for: Molecular characterization of subcutaneous panniculitis-like T-cell lymphoma reveals upregulation of immunosuppression- and autoimmunity-associated genes
Source: Orphanet J Rare Dis. 2014 Nov 12;9:160. doi: 10.1186/s13023-014-0160-2 (PMC4320460; doi:10.1186/s13023-014-0160-2)

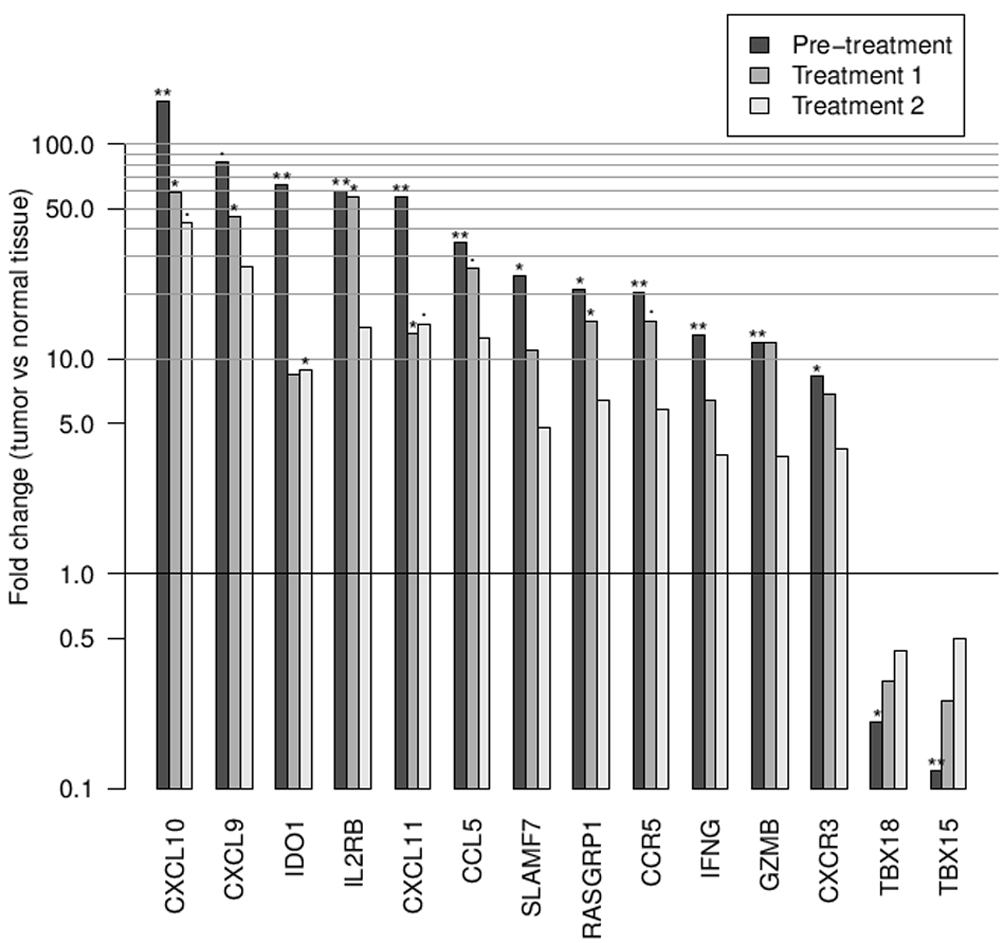

Supplement: Additional file 1: — SPTL gene expression profile changes during treatment. The SPTL skin lesions show changes in the expression of up regulated genes during ongoing clinical response to systemic steroid (40-60 mg/d) and methotrexate (10 mg/week). The bars are presented on logarithmic scale indicating the microarray fold changes of SPTL samples compared to normal subcutaneous fat tissue (n=2). Pre-treatment, treatment1, and -2 values are based on three, three, and two patients, respectively. The gray horizontal line indicates neutral expression level with fold change of one. Statistical significance between SPTL samples and normal subcutaneous fat tissue is indicated with ** (p<0.01), * (p<0.05) and “.” (p<0.1). [file 13023_2014_160_MOESM1_ESM.tiff]
